# A comparison of field portable X-ray fluorescence (FP XRF) and inductively coupled plasma mass spectrometry (ICP-MS) for analysis of metals in the soil and ambient air

**DOI:** 10.21203/rs.3.rs-3849271/v1

**Published:** 2024-01-11

**Authors:** Kelsey Schmidt, Daniel Autenrieth, Raja Nagisetty

**Affiliations:** Montana Technological University; Montana Technological University; Montana Technological University

**Keywords:** lead, arsenic, correction factor, level of agreement, Superfund

## Abstract

When analyzing metal concentrations in the soil and ambient air, accurate and reliable results are essential. Inductively coupled plasma mass spectrometry (ICP-MS) is considered the benchmark analytical method for environmental soil and air filter samples containing metals. Field portable X-ray fluorescence (FP XRF) can provide more timely results with lower ongoing costs, but the results are not as accurate as ICP-MS. The primary goal of this study was to find an optimal method to maximize the level of agreement between FP XRF results and ICP-MS results when analyzing metal concentrations in soil and ambient air samples in a U.S. Superfund community. Two different correction factor methods were tested to improve the prediction of ICP-MS concentrations using FP XRF for arsenic and lead in soil and ambient air. Ninety-one residential soil samples and 42 ambient air filter samples were analyzed in a split-half design, where half the samples were used to create the correction factors and the other half to evaluate the level of agreement between the analytical methods following FP XRF correction. Paired t-tests, linear regression plots, and Bland-Altman plots were utilized to examine which correction factor provided the highest level of agreement between the two methods. Based on the results from this study, it was determined that a ratio correction factor method provided the best fit for this FP XRF analytical device.

## Introduction

Butte, Montana, known as the “Richest Hill on Earth”, is home to over 100 years of mining, milling, and smelting activity that produced approximately 32 billion pounds of metals, including copper, zinc, manganese, lead, molybdenum, gold, and silver (Duaime et al., 2004). Surrounded on three sides by the Continental Divide, the town has just over 30,000 residents and sits 5,500 feet above sea level (U.S. Census Bureau, 2019). Today, Butte and the surrounding area make up the largest Superfund complex in the United States due to the extensive amount of mine waste created during historic mining activity (Nagisetty et al., 2020). One of the primary environmental concerns regarding this mine waste is the widespread air and soil contamination, consisting of heavy metals such as lead, arsenic, mercury, and cadmium. These contaminants are associated with harmful short-term and long-term health effects including respiratory diseases, reproductive issues, and various cancers (Maret and Sandstead, 2006, O’Neal, 2015, Pohanka, 2019, Huat et al., 2019, Jomova et al., 2011, Zahir et al., 2005, Godt et al., 2006). Unlike most other superfund sites in the United States, open-pit mining continues at present on the northeast side of Butte, adjacent to a residential area. A previous study in Butte found statistically higher (α=0.05) concentrations of Al, As, Cd, Cu, Mn, Mo, and U in hair samples of residents in Butte when compared to residents in a nearby county with no history of mining (Hailer et al., 2017). Additionally, there were elevated concentrations of Cu, Zn, and Pb in soil samples and elevated As and Mn in the ambient air when compared to typical US ambient air concentrations (Hailer et al., 2017).

When determining the concentration of metals in the air and soil, accurate and reliable results are essential. Traditionally, when environmental samples are collected for metal analysis, inductively coupled plasma mass spectrometry (ICP-MS) is used to quantify metal concentrations (Creed et al., 1994). ICP-MS utilizes argon plasma to dissociate the molecules in a sample and form charged ions (Thomas, 2001). These charged ions are then analyzed using a mass spectrometer, which determines element type and quantity based on the mass and charge of each ion (Houk et al., 1980). While sample analysis costs both time and money, ICP-MS is a desirable analytical method because it can detect a multitude of elements simultaneously with a low limit of detection and low limit of quantification (Bulska and Wagner, 2016). Additionally, sample preparation is quick and straightforward compared to other methods when following EPA method 6020A (Wilschefski and Baxter, 2019). Unlike ICP-MS, field-portable X-ray fluorescence (FP XRF) uses an X-ray beam to displace electrons in the smallest orbital within atoms (Jenkins et al., 1995). This electron displacement causes the atom to lose energy, and when measured, the lost energy can determine the type of element in the sample as well as the quantity (Jenkins et al., 1995). In recent years, XRF has been developed by companies like Thermo Fisher and Bruker as a portable device that can be used in a field setting instead of a lab (Bosco, 2016). This design allows for quicker direct-reading results of a variety of sample types, including dusts, soils, paints, and air filters.

FP XRF has the capability to deliver results in close to real-time, eliminating the need for laboratory analysis, and making this device beneficial in both environmental and occupational settings. A previous study compared these two methods for the analysis of lead in environmental soil surrounding a large lead smelter in South Australia (Maliki et al., 2017). When using the Student’s paired t-test, the authors found no statistically significant difference between reported mean ICP-MS and FP XRF derived concentrations at the 5% probability level (Maliki et al., 2017). A similar comparison study of inductively coupled plasma optical emission spectrometry (ICP-OES) and XRF for lead and arsenic in soil samples was completed in Chihuahua City Mexico, which is known for its heavy metal pollution from years of metal production and refining (Delgado et al., 2011). This study found no statistically significant differences among the arsenic concentrations but did find a statistically significant difference between the two techniques for lead (Delgado et al., 2011). A study in 2019 compared inductively coupled plasma atomic emission spectrometry (ICP-AES) to XRF when analyzing manganese fume in a metal casting foundry (Davis, 2019). The author calculated a p-value of 0.162 after completing a one-sample Wilcoxon test, concluding that there was no statistically significant difference in means between these concentrations (Davis, 2019).

### Objectives

The primary goal of this study is to evaluate the level of agreement between contaminant concentrations in environmental samples analyzed via FP XRF in comparison with results obtained from laboratory ICP-MS analysis. These contaminants include arsenic and lead in the soil along with arsenic, copper, lead, manganese, molybdenum, and zinc in the ambient air. This goal will be addressed through three research objectives. First, calibration factors will be developed for each metal and applied to the XRF device in order to assess the strength of the associations between metal concentrations using FP XRF and ICP-MS. Next, correction factors will be established to predict ICP-MS concentrations given XRF concentrations using two different methods. Finally, the level of agreement between the corrected FP XRF concentrations and the ICP-MS concentrations will be evaluated to determine the best possible correction method.

## Methods and materials

### Soil Sampling

**Figure 1:**
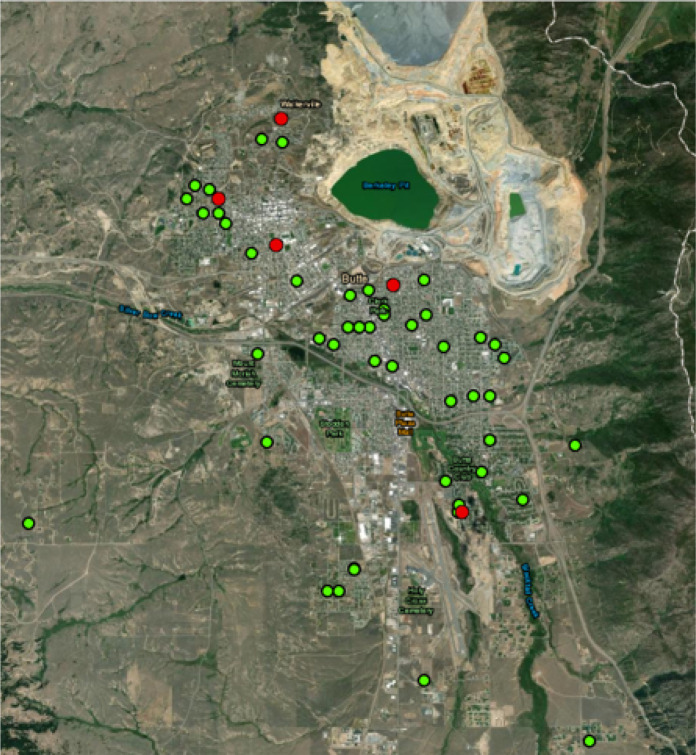
Soil and air sampling locations in Butte, MT. Air sampling locations are seen in red (n=5) and soil sampling locations are seen in green (n=46).

Between October and December 2020, following a modified method from the Montana Department of Environmental Quality (MT-DEQ, 2013), 91 soil samples were collected in residential yards throughout Butte, MT, shown in Figure 1, following an IRB approved protocol as part of a larger study. Unnecessary vegetation was removed and soil was collected from 0 to 6 inches deep using a sanitized hand trowel. A sample was taken from the middle of each yard, equidistant from the residence and yard perimeter, and combined with a sample from the dripline into a collection bag. Samples were obtained from the front and back yard of each residence, totaling two combined samples from each location. Soil was also sampled from gardens when applicable. In the lab, soil was exposed to the atmosphere for 24 hours without heat in order to dry. All samples were sieved to a size of 250 μm and smaller. Half of each sample was prepared for XRF analysis according to the manufacturer recommended method for standard soil sampling (Niton, 2004) and analyzed for 80 source seconds. The remaining half of each sample was analyzed by an independent lab using ICP-MS for arsenic and lead following EPA method 6020A (EPA, 1998).

### Air Sampling

Air sampling was conducted at five locations throughout Butte over seven weeks during October and November of 2020, shown in Figure 1. Each location utilized an Institute of Medicine (IOM) air sampler to collect the inhalable fraction (50% cut point of 100 μm aerodynamic diameter) of ambient air particulate matter (PM). The 50% cut point mentioned previously refers to the PM collection efficiency of each air sampler. PM larger than 100 μm is collected on the filter with less than 50% efficiency while PM smaller than 100 μm is collected at a higher efficiency rate than 50% (McKenzie et al., 1982). This cut point was selected because it accurately represents particulate matter than can be inhaled into the nasal passageways and upper respiratory tract. Smaller particles can travel further into the lungs and reach the gas exchange region (Millage et al., 2010). The air sampling station located nearest to the active mine had duplicate air samplers and the remaining sites had one each. IOM air samplers were placed 5 feet above the ground to measure PM concentrations at approximate breathing zone height. Air was sampled at 2 L/m for one week at a time with co-located field blanks present at the location nearest the active mine, following the established SKC IOM method (Kenny et al., 2001). Filters were changed every seven days for six consecutive weeks, totaling 42 filters available for analysis. Particulate was collected on 25mm PVC filters that were desiccated and pre-weighed before use. After sampling, each filter was dried and post-weighed for gravimetric analysis. Samples were analyzed by a FP XRF device for 80 source seconds using the manufacturer’s recommended analytical method and concentrations were corrected accordingly based on the calibration factors established using known concentrations (Niton, 2004). Following XRF analysis, samples were digested, diluted, filtered and analyzed using ICP-MS for 2 elements according to the EPA method 6020A protocol (EPA, 1998).

### Statistical Analysis

#### FP XRF Calibration

In order to accurately compare metal concentrations between ICP-MS and XRF, calibration factors were created for the FP XRF device. Three National Institute of Standards and Technology (NIST) soil standards, low standard 2709 San Joaquin soil, medium standard TILL-4p soil, and high standard 2710 Montana Soil, were analyzed ten times along with a laboratory blank for 80 source seconds (Mackey et al., 2010). For each of the contaminants, known metal concentrations were plotted against measured concentrations from the XRF to produce a calibration curve and linear regression equation based on this relationship. NIST calibration values were treated as the predictor (X) variable and the measured concentrations from XRF were considered the response (Y) variable. The inverse regression equation for each metal was utilized to correct all soil and air concentrations from the XRF, creating the calibrated concentrations used below.

#### Comparison of ICP-MS Concentrations to Calibrated FP XRF Concentrations

To assess the first research objective regarding calibration factors and the corresponding strength of associations, Spearman rank-order correlational analysis was performed between the ICP-MS and calibrated FP XRF concentrations for arsenic and lead. Log_10_ transformation was explored for all contaminant concentrations, as they were not normally distributed when determined using the Anderson-Darling test for normality. Upon log_10_ transformation of the lead and arsenic data, it was determined that they were then normally distributed.

For the second research objective, each pair of data, including one ICP-MS result and the corresponding FP XRF result, were assigned a random number. Half of the pairs for each metal were selected using the highest half of the random numbers. This half of the data set, deemed the model data, was utilized to create a correction equation for the remaining data. Similar to the calibration equations developed above, the model pairs were plotted against each other to create a linear regression model. ICP-MS values were treated as the predictor variable (X) and concentrations measured by FP XRF were treated as the response (Y) variable. The inverse regression equation established from the model was used as the first correction equation. This equation was used on the remaining pairs of data, deemed the test data set, to predict the expected ICP-MS concentrations. In addition to the development of a correction factor based on linear regression, a ratio of the means (mean of ICP-MS concentrations divided by the mean of FP XRF concentrations) of the model data was calculated and applied to the remaining test FP XRF concentrations as well.

**Figure 2:**
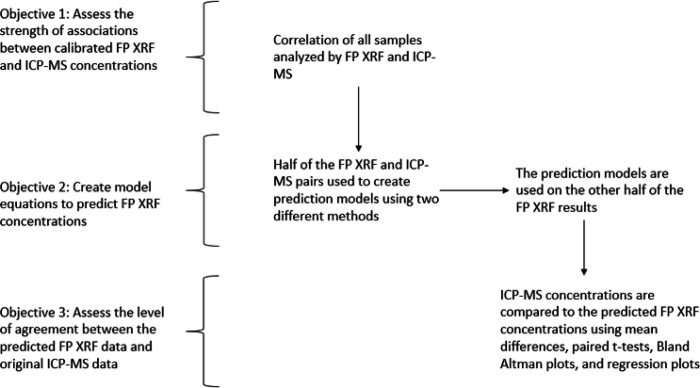
Experimental design flow chart of the statistical analyses in this study

To address the third and final research objective, both the regression corrected and ratio corrected data, along with the uncorrected FP XRF data and the ICP-MS test data, were compared using box and whisker plots. Paired t-tests comparing the mean concentration of each metal using both correction methods to the reference ICP-MS test data were employed to obtain p-values. Normality of the mean differences was assessed using an Anderson-Darling test. Additionally, qualitative Bland-Altman plots of the untransformed concentrations were developed to compare the limits of agreement between the different correction methods for each metal. From there, based on the statistical analyses described above, the correction factor that provided the highest level of agreement between predicted test concentrations when compared to the test ICP-MS data was selected and applied to the remaining test data. A flowchart of the experimental design for this study is seen in Figure 2.

The level of statistical significance for all analyses was set at α=0.05. All statistical analyses in this study were performed using Minitab Statistical Software version 19 (State College, PA, USA).

#### Comparison of ICP-MS Air Filter Concentrations to Raw FP XRF Air Filter Concentrations

Originally, the statistical analyses used to evaluate the soil samples in this study were also going to be utilized for the ambient air data. Due to inconclusive results from both the FP XRF concentrations and the results from the benchmark ICP-MS method, the analyses discussed above could not be completed on the air data collected. Of the 42 air samples obtained, only 5 paired samples had concentrations above the limit of detection when analyzed using FP XRF and ICP-MS. In order to ensure an adequate number of samples to run the statistical analyses discussed above, a dust dispersion chamber was utilized to collect more air filter samples with a detectable concentration of metals.

## Results

A total of 91 soil samples were collected over the course of 3 months. For arsenic, samples ranged from 9.59 – 97.53 mg/kg and 6.00 – 95.00 mg/kg for FP XRF and ICP-MS, respectively. Samples ranged from 24.75 – 2779.77 mg/kg and 15.00 – 2560.00 mg/kg for lead from the FP XRF and ICP-MS, respectively. Of the 91 samples collected, 81 pairs were above the LOD (1.00 mg/kg) for arsenic and all were above the LOD (3.00 mg/kg) for lead. Paired concentrations below the LOD were not included in the statistical analyses. To satisfy the first objective of this research, correlational strength was determined between the calibrated FP XRF values and corresponding ICP-MS data, shown in Figure 2. Both metals demonstrated a strong, positive correlation with Spearman coefficients of 0.850 and 0.981 for arsenic and lead, respectively. Moreover, arsenic had an R-squared value of 58.3% and lead presented an R-squared value of 97.3% during regression analysis.

Descriptive statistics of the calibrated FP XRF and ICP-MS test data along with the data from each correction factor method are summarized in Table 1. The arithmetic mean for arsenic concentrations analyzed by ICP-MS was 42.2 mg/kg and 49.7 mg/kg for the uncorrected, calibrated FP XRF concentrations. Arithmetic means of 48.5 mg/kg and 39.4 mg/kg were calculated for the regression corrected and ratio corrected concentrations, respectively. The geometric mean for arsenic concentrations analyzed by ICP-MS was 37.5 mg/kg and 46.7 mg/kg for the calibrated uncorrected concentrations analyzed by FP XRF. The geometric means for FP XRF concentrations were 38.7 mg/kg and 37.1 mg/kg when the regression correction factor and ratio correction factor were applied, respectively.

For lead, the arithmetic mean concentrations analyzed by ICP-MS was 365.4 mg/kg and 335.7 mg/kg for the uncorrected, calibrated FP XRF concentrations. Arithmetic means of 430.0 mg/kg and 358.4 mg/kg were calculated for the regression corrected and ratio corrected concentrations, respectively. The geometric mean of ICP-MS concentrations was 149.4 mg/kg and 153.8 mg/kg for the uncorrected, calibrated FP XRF concentrations. Geometric means for FP XRF concentrations were 155.2 mg/kg when corrected using a regression method and 164.2 mg/kg when corrected using a ratio method. The correction factor equations for each metal are shown in Table 2. The regression corrected method utilizes log base 10 values because both arsenic and lead concentrations were not normally distributed before log-transformation. The calibration equations calculated from known standards are also provided in Table 2.

**Figure 3:**
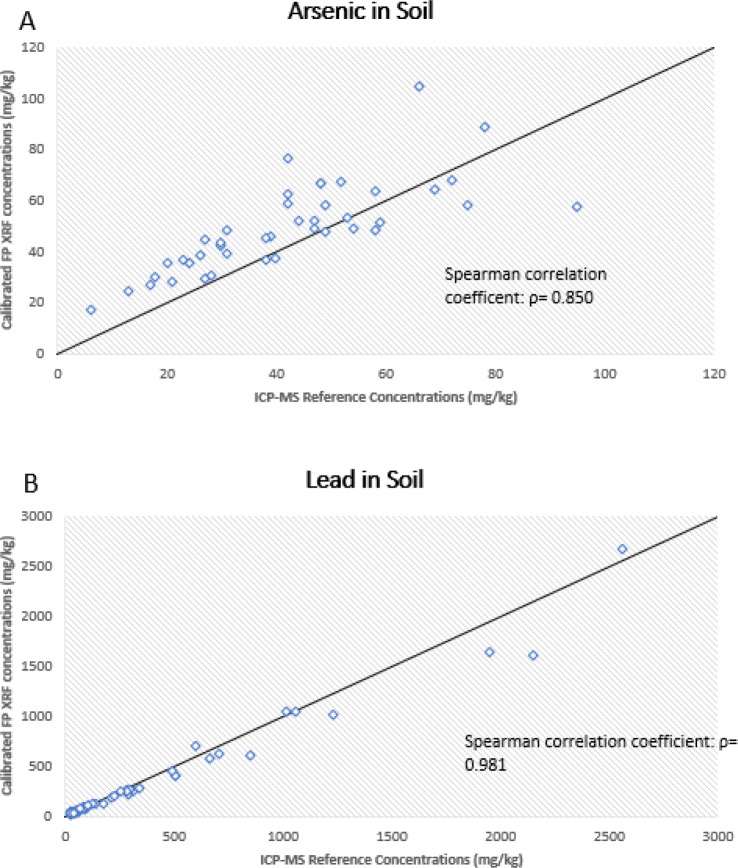
The association between (A) arsenic (n=81) and (B) lead (n=91) in soil obtained by ICP-MS and calibrated FP XRF

Box and whisker plots for arsenic and lead are shown in Figure 3. The reference test data set, analyzed by ICP-MS, was compared to FP XRF concentrations corrected using a linear regression model and a ratio of the mean concentrations. Both sets of corrected concentrations were compared to the ICP-MS data using paired t-tests. Normality was measured using an Anderson-Darling test and it was determined that none of the mean differences for either metal were normally distributed. For arsenic, log_10_ transformation improved normality of the uncorrected mean differences, regression corrected mean differences, and ratio corrected mean differences. When log_10_ transformation was employed for the lead data sets, normality improved for the mean differences of the regression corrected data set but it was determined that the uncorrected FP XRF mean differences and ratio corrected mean differences were not log-normally distributed. The p-values from these paired t-tests and mean differences between concentrations are shown in Table 3. For arsenic, p-values of 0.123 and 0.152 were calculated for the regression corrected and ratio corrected concentrations, respectively. The mean difference between ICP-MS and regression corrected FP XRF concentrations was 6.22 mg/kg. When compared to ICP-MS, the ratio corrected data had a mean difference of 2.81 mg/kg. Lead concentrations presented a similar pattern when compared to the ICP-MS data. P-values of 0.061 and 0.585 were calculated from paired t-tests for regression corrected FP XRF concentrations and ratio corrected concentrations, respectively. The regression corrected data for lead presented a mean difference of −64.5 mg/kg and the ratio corrected FP XRF concentrations had a mean difference of 7.2 mg/kg when compared to the ICP-MS reference data.

Of the 42 air filters analyzed, only 5 filters had detectable levels of either arsenic or lead. Due to the lack of arsenic and lead detection, the statistical analyses conducted for soil could not be applied to the air data. Of the 5 filters with detectable arsenic and lead concentrations, none came close to any federal or state action levels or regulations for air quality. The air sampling results from this research are consistent with other air quality studies conducted in Butte, MT (Hailer et al., 2017, Montana Resources LLP, 2019).

**Figure 4:**
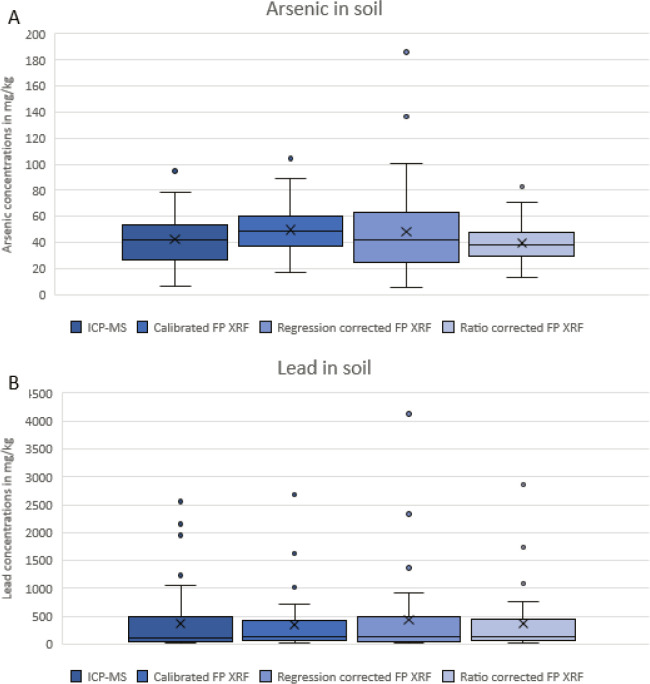
Box and whisker plots showing arsenic (a) and lead (b) concentrations (mg/kg) measured by ICP-MS, the calibrated FP XRF device, and FP XRF concentrations after the inverse regression equations and ratio equations were applied to the data. The box represents the 25^th^-75^th^ percentiles; the centerline represents the median concentration; the X represents the mean and the circles represent outliers which are defined as concentrations that were 1.5 times the interquartile range above the third quartile.

Qualitative Bland-Altman plots using untransformed data for both metals are provided in Figure 4. The mean differences and 95% limits of agreement, along with a 95% confidence interval, are shown in each plot. For arsenic, the agreement interval width between ICP-MS and the calibrated FP XRF concentrations was 49.94. When comparing ICP-MS to the regression corrected and ratio corrected arsenic concentrations using Bland-Altman plots, the agreement interval widths were 100.20 and 48.88, respectively. In regards to lead, the agreement interval widths displayed a similar pattern. For ICP-MS lead concentrations compared to calibrated FP XRF concentrations, an agreement interval width of 391.06 was calculated. The agreement interval increased for the regression corrected data set to 941.60. When intervals were calculated for the ratio corrected lead data, the width was 361.78.

## Discussion

This study found that lead and arsenic concentrations analyzed via both methods were fairly comparable after the addition of a ratio correction factor, as determined by mean differences and standard deviations of the data. Before the addition of a correction factor, arsenic was overestimated and lead was underestimated by FP XRF, respectively, when compared to the ICP-MS data. Three research objectives were employed to determine which correction method provided the highest level of agreement when analyzing arsenic and lead using FP XRF by comparing concentrations to the benchmark analytical method, ICP-MS. The first research objective was to assess the strength of association between calibrated FP XRF data and paired ICP-MS data. This research found that there was a relatively strong positive correlation between both analytical methods for lead and arsenic before any additional correction factor was applied. An R-squared value of 97.3% was obtained for lead with a Spearman coefficient of 0.981. Arsenic had an R-squared value of 58.3% and a Spearman coefficient of 0.850 when the calibrated FP XRF data was compared to the paired test ICP-MS values. Overall, FP XRF had a tendency to underestimate lead concentrations and overestimate arsenic concentrations. A previous study completed by Wu et al. showed similar results when comparing an FP XRF device to ICP-AES. There was a higher level of agreement between both analytical methods for lead concentrations but considerable variability when looking at arsenic. Additionally, they determined that FP XRF overestimated arsenic concentrations, similar to this study (Wu et al., 2012). In order to improve the level of agreement for this device, different correction methods were utilized to determine which would compensate best for the underestimation and overestimation of lead and arsenic, respectively.

To satisfy the second and third research objectives, two different correction factors were calculated and applied to the FP XRF soil concentrations. The first correction method utilized an inverse linear regression equation derived from a portion of the ICP-MS data. The second correction method used a ratio of the mean ICP-MS model data divided by the mean FP XRF model data that was applied to the calibrated FP XRF concentrations. Based on the quantitative and qualitative analyses conducted in this study, we demonstrated that the application of a ratio correction factor provides the most accurate fit for this FP XRF device for both arsenic and lead. When compared to the ICP-MS reference concentrations, the regression corrected data for both metals was not significantly different based on the p-values from paired t-tests. For arsenic, mean differences between concentrations decreased from −6.22 mg/kg for the regression corrected data to 2.81 mg/kg for the ratio corrected FP XRF concentrations. The mean difference between concentrations for lead increased from 29.7 mg/kg with calibrated XRF values to −64.5 mg/kg when the inverse regression equation was applied. Unlike the regression correction factor, mean differences between concentrations decreased, from 29.7 mg/kg to 7.0 mg/kg, when the ratio correction factor was applied. Moreover, the p-values from paired t-test improved dramatically. When compared to ICP-MS, the calibrated FP XRF concentrations for arsenic had a statistically significant p-value of 0.000 before a correction factor was applied. The regression corrected data and ratio corrected data had non-statistically significant p-values of 0.123 and 0.152, respectively. For lead, the calibrated FP XRF data when compared to ICP-MS had a statistically significant p-value of 0.039 and the regression corrected data had a p-value of 0.061. The level of agreement increased when the ratio correction factor was applied to FP XRF concentrations, with a p-value of 0.585.

The Bland Altman plots created for this study provide a visual tool to help determine which correction factor provides the best fit for future FP XRF data. The range between upper and lower limits of agreement decreased from 49.94 mg/kg to 48.88 mg/kg and from 391.06 mg/kg to 361.78 mg/kg for the ratio corrected data from arsenic and lead, respectively. When the regression correction factor was applied for lead, both LOA’s actually increased along with the mean difference between concentrations. There was a visible fanning pattern among all data points for both metals as measured concentrations increase. This pattern suggests that higher concentrations of arsenic and lead measured by the FP XRF tend to have a lower level of agreement when compared to the benchmark analytical method, ICP-MS.

### Limitations

Limitations for this study include deviation from EPA method 6200 for FP XRF analysis, namely in drying the soil samples. EPA method 6200 recommends drying all soil samples with heat using an oven. All samples in this study were air dried to avoid possible mercury exposure, as mercury can become volatile when heated. Moreover, the soil sampling method followed from the Montana Department of Environmental Quality (MT-DEQ, 2013) was modified for this research. This method recommends soil collection from 0 to 12 inches deep to get an accurate measure of all metals. Since this study was solely focused on arsenic and lead, soil was only collected from 0 to 6 inches deep as these two metals tend to be concentrated in the upper portion of the ground. During the 6 weeks of air sampling conducted for this research, approximately 3 of these weeks had snow on the ground. It is possible that the snow prevented the aerosolization of arsenic and lead in the ambient air, resulting in lower concentrations of these two metals collected on the air filters.

## Conclusions

This study determined that when measuring arsenic and lead concentrations in soil, FP XRF technology tends to overestimate arsenic concentrations and underestimate lead. Even with this decrease in accuracy, the FP XRF device used for this research demonstrated a fairly strong level of agreement with ICP-MS before any correction factor was applied, especially for lead. The application of an inverse linear regression correction factor slightly improved the arsenic data but decreased the level of agreement for lead concentrations. It was determined that a ratio correction factor provided the best fit for this portable device for both metals. These results suggest the importance of a correction factor when measuring metal concentrations in environmental samples. Additionally, they demonstrate that FP XRF has the potential to accurately measure metal samples once a correction factor has been applied, making this a desirable method over ICP-MS. Not only is FP XRF non-destructive, it delivers results in real time. This would greatly decrease analytical delays when waiting for ICP-MS results from labs.

The results from this study demonstrated that the addition of a ratio correction factor improves the overall accuracy of this FP XRF for environmental soil samples. The next step in this research is to compare both analytical methods for air filter samples using a dust dispersion chamber to apply detectable concentrations of metals on these filters, simulating the collection of metals in ambient air. Additionally, collecting more soil samples with higher concentrations of arsenic and lead would be beneficial to better determine if a ratio correction factor would still be the best fit for this FP XRF device due to decreased inaccuracy at higher concentrations when compared to ICP-MS.

Ideally, this same correction factor method could be applied to other FP XRF devices as well. As part of a larger study involving community engagement, FP XRF could potentially be utilized by members of the community to analyze their own soil samples from yards, gardens, parks, etc.

## Figures and Tables

**Figure 5:**
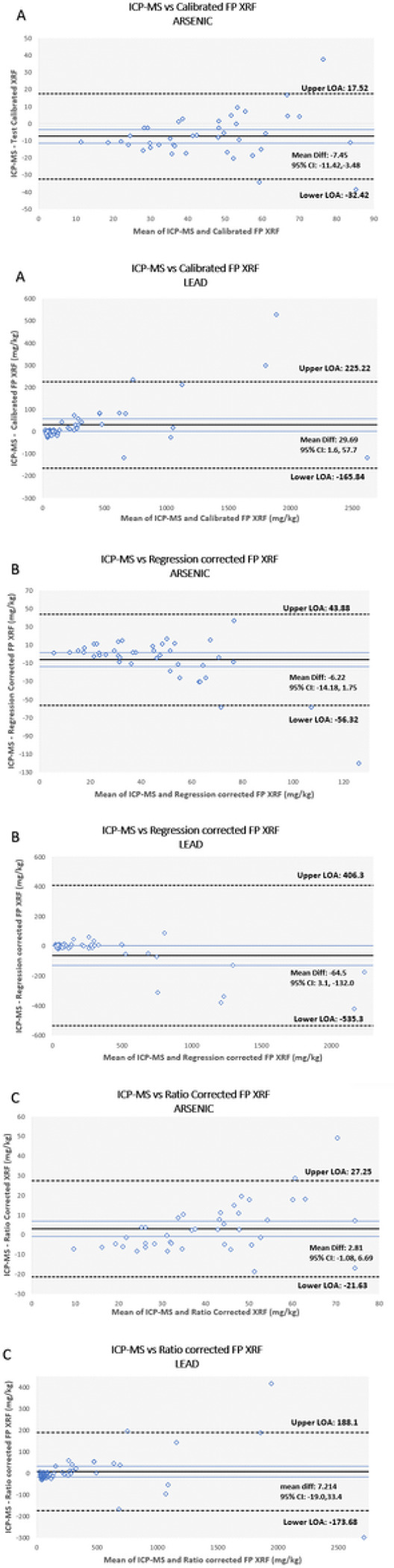
Bland and Altman plots of the differences between lead and arsenic concentrations measured by ICP-MS and calibrated FP XRF (y-axis) vs. the mean of the two instrument measurements (x-axis). Corrected FP XRF concentrations using the inverse regression method are shown in plots B and concentrations using the ratio correction method are shown in plots C. The solid black line represents the mean difference between both concentrations, the surround blue lines represent the 95% confidence interval, and the dashed black lines represent the limits of agreement.

**Table 1:** Summarized statistics of (A) arsenic and (B) lead concentrations (mg/kg) in soil measured by ICP-MS and both uncorrected and corrected concentrations measured using FP XRF.

Arsenic (mg/kg)				

A		FP XRF		

	ICP-MS- Reference Concentrations	Calibrated FP XRF	Regression Corrected	Ratio Corrected

Arithmetic Mean (+/− SD)	42.2 (± 19.3)	49.7 (± 17.5)	48.5 (± 34.5)	39.4 (± 13.9)

Geometric mean (+/− GSD)	37.5 (± 1.7)	46.7 (± 1.4)	38.7 (± 2.0)	37.1 (± 1.4)

Range	6.0 – 95.0	16.9 – 104.5	13.6 – 185.9	13.4 – 82.9

Coefficent of Variation	47.0	35.2	71.3	35.2
**Lead (mg/kg)**				

B		FP XRF		

	ICP-MS- Reference Concentrations	Calibrated FP XRF	Regression Corrected	Ratio Corrected

Arithmetic Mean (±SD)	365.4 (± 556.4)	335.7 (± 508.4)	430.0 (± 756.0)	358.4 (± 542.7)

Geometric Mean (± GSD)	149.4 (± 3.8)	153.8 (± 3.4)	155.2 (± 4.0)	164.2 (± 3.4)

Range	24.0 – 2560.0	19.7 – 2679.0	14.7 – 4135.3	21.0 – 2859.8

Coefficent of Variation	152.3	151.4	176.0	151.4

**Table 2:** Calibration and correction factors for lead and arsenic in soil.

Calibration & Correction Factors			

	Calibration Equation	Regression Correction Factor	Ratio Correction Factor

Arsenic	x= (y+7.373)/1.004	log(x)=(log(y)-0.8563)/0.5124	36.82/48.82=0.754

Lead	x= (y-4.335)/1.036	log(x)= (log(y)-0.2798)/0.8705	205.30/206.48=0.994

**Table 3:** P-values from paired t-tests (α=0.05) and mean differences between ICP-MS, calibrated FP XRF, and FP XRF concentrations after both correction factors were applied to arsenic (a) and lead (b).

A	ARSENIC		
	Comparisons with ICP-MS reference concentrations			

Method	Mean difference (95% CI)	Mean log difference (95%)	P-value

Calibrated FP XRF	−7.45 (−11.42, −3.48)	−0.096 (−0.135, −0.058)	0.000
Regresson Corrected XRF	−6.22 (−14.18, 1.75)	−0.014 (−0.062, 0.034)	0.123
Ratio Corrected XRF	2.81 (−1.08, 6.69)	0.004 (−0.034, 0.043)	0.152

**B**	**LEAD**		
	**Comparison with ICP-MS reference concentrations**			

Method	Mean difference (95% CI)	Mean log differerce (95% CI)	P-value

Calibrated FP XRF	29.7 (1.6, 57.7)	−0.013 (−0.042, 0.017)	0.039
Regresson Corrected XRF	−64.5 (−132.0, 3.1)	−0.017 (−0.044, 0.011)	0.061
Ratio Corrected XRF	7.0 (−18.7, 32.7)	−0.041 (−0.071, −0.011)	0.585
